# Soybean Oil Replacement by Palm Fatty Acid Distillate in Broiler Chicken Diets: Fat Digestibility and Lipid-Class Content along the Intestinal Tract

**DOI:** 10.3390/ani11041035

**Published:** 2021-04-06

**Authors:** Beatriz Jimenez-Moya, Ana C. Barroeta, Alba Tres, María Dolores Soler, Roser Sala

**Affiliations:** 1 Animal Nutrition and Welfare Service (SNiBA), Animal and Food Science Department, Faculty of Veterinary, Universitat Autònoma de Barcelona, Edifici V, Travessera dels Turons, 08193 Bellaterra, Spain; Beatriz.Jimenez@uab.cat (B.J.-M.); Ana.Barroeta@uab.cat (A.C.B.); 2 Departament de Nutrició, Ciències de l’Alimentació i Gastronomia, Campus de l’Alimentació Torribera, Facultat de Farmàcia i Ciències de l’Alimentació, Universitat de Barcelona, Av Prat de la Riba, 171, 08921 Santa Coloma de Gramenet, Spain; atres@ub.edu; 3 Institut de Recerca en Nutrició i Seguretat Alimentària (INSA-UB), Universitat de Barcelona, Av Prat de la Riba, 171, 08921 Santa Coloma de Gramenet, Spain; 4 AviFeed Science, Department of Animal Production and Health Public Veterinary Health and Food Science and Technology, Facultad de Veterinaria, Universidad Cardenal Herrera-CEU, CEU Universities, Calle Tirant lo Blanch, 7, 46115 Alfara del Patriarca, Valencia, Spain; mariola@uchceu.es

**Keywords:** fat digestibility, lipid classes, free fatty acid, fat by-product, fatty acid distillate, alternative energy source, broiler chickens, poultry, intestinal tract

## Abstract

**Simple Summary:**

Palm fatty acid distillate is a by-product of palm oil refining. It is of both environmental and economic interest to include it in the diets of broiler chickens. However, its high saturation degree and acidity level limit its use. This study aimed to assess the effect of replacing soybean oil with increasing levels of palm fatty acid distillate on the utilization of fat by broilers. Dietary fat hydrolysis was mostly affected by the age of the bird and including palm fatty acid distillate mainly affected the absorption process. The replacement of soybean oil by palm fatty acid distillate reduced the total fat utilization, and in starter chicks delayed the site of fatty acid absorption. As the age increased, the digestibility of saturated fatty acids improved, and, above all, it improved the free fatty acid utilization. Therefore, the potential inclusion of palm fatty acid distillate for broiler feeds depends on the age of the bird. It would not be recommended to include this by-product in starter feeds. However, for the grower-finisher phase, blending palm fatty acid distillate with soybean oil (1:3, w/w) could be a suitable alternative, that does not have negative repercussions for either fatty acid absorption or growth performance.

**Abstract:**

Palm fatty acid distillate (PFAD) is a by-product of palm oil (P) refining. Its use in chicken diets is a way to reduce the cost of feed and the environmental impact. Its low unsaturated:saturated fatty acid ratio (UFA:SFA) and its high free fatty acid (FFA) level could be partially counteracted by its blending with soybean oil (S). The objective was to assess the effect of replacing S with different levels of PFAD on lipid-class content and fatty acid (FA) digestibility along the intestinal tract and in the excreta of 11 and 35-day-old broiler chickens. Five experimental diets were prepared by supplementing a basal diet with S (S6), PFAD (PA6), two blends of them (S4-PA2 and S2-PA4), or P (P6) at 6%. Replacing S with PFAD did not affect performance parameters (*p* > 0.05) but negatively affected feed AME, FA digestibility, and FFA intestinal content (*p* < 0.05), especially in starter chicks. Including PFAD delayed total FA (TFA) absorption (*p* < 0.05) at 11 days, but at 35 days it did not affect the TFA absorption rate. The use of PFAD blended with S, when FFA ≤ 30% and UFA:SFA ≥ 2.6, led to adequate energy utilization in broiler grower-finisher diets.

## 1. Introduction

Fats are usually used in poultry diets as they satisfy a large fraction of the energy requirements. Palm fatty acid distillate (PFAD) is a fat by-product from the production of refined palm oil (P) which is one of the most produced and consumed vegetable oils worldwide [1]. Usually, P is obtained by a physical refining process that includes different steps, namely degumming, winterization (optional), bleaching, and deodorization [2]. The latter step is a vacuum steam distillation process that removes the FFA that are accumulated in the fatty acid distillate [3]. PFAD is characterized by having a high proportion of free fatty acids (FFA: 87–94%, being rich in saturated FA (SFA) and including other compounds such as tocopherols [4]). Based on a circular economy and taking into account the rising cost of conventional fats, there is increased interest in upcycling by-products from the fat industry for animal feeding to reduce the cost of feed formulation and also the environmental impact [5,6].

Assessing the digestibility of a fat ingredient is one of the clearest ways of defining its nutritional value for an animal. Conventional fat and oil sources used in poultry feed mainly consist of triacylglycerol (TAG) molecules. During digestion, TAGs, and diacylglycerols (DAGs) are hydrolyzed into monoacylglycerol (MAG) and FFA, which are incorporated into dietary mixed micelles (DMM) to attain the enterocytes for their absorption. Therefore, studying the evolution of the lipid classes (TAG, DAG, MAG, and FFA) and FA digestibility along the different segments of the gastrointestinal tract (GIT) may be of great interest for understanding the dynamics of fat digestion [7,8], mainly in new alternative fat sources rich in FFA and also in fat blends.

The PFAD is rich in FFA and also in SFA. It is well known that the degree of FA saturation plays and important role in fat absorption. In broiler chickens, although SFAs are not digested as well as unsaturated FA (UFA), several authors have found a synergistic effect when saturated sources are blended with unsaturated ones [9,10,11]. In fact, recent studies have found that the saturation degree of the dietary fat has more influence on fat digestibility than its FFA content [5,12]. Moreover, it has been suggested that there is a positive effect on FFA digestibility when there are increasing amounts of DAG or MAG, because their emulsifying effect enhances the inclusion of FFA in DMM [13]. However, there are few studies on FA absorption using blends of crude (rich in TAG) and acid (rich in FFA) oils.

Furthermore, it is accepted that the absorption of FA is also affected by the age of the chickens. Better results in the hydrolysis–absorption process along with the GIT of conventional and alternative fats have been obtained in grower-finisher chickens compared to starter broiler chickens [8].

Therefore, our hypothesis is that PFAD in combination with soybean oil (S) could be considered as an alternative energy source for broiler chicken diets, but the use of PFAD might be influenced by the age of the chicken. Thus, the aim of the present study was to research the effect of replacing S with graded levels of PFAD on lipid-class content and FA digestibility along the intestinal segments of the GIT (upper and lower jejunum, upper and lower ileum) and in the excreta in starter and grower-finisher broiler chickens.

## 2. Materials and Methods

### 2.1. Housing and Animals

The study was carried out at the animal experimental facilities of the Servei de Granges i Camps Experimentals (Universitat Autònoma de Barcelona; Bellaterra, Barcelona, Spain). All management practices and procedures were approved by the Animal Ethics Committee (CEEAH) of the same institution (number code: 3938), in accordance with the European Union guidelines for the care and use of animals in research (2010/63/EU).

A total of 480 newly hatched female broiler chickens (Ross 308) were obtained from a commercial hatchery (Pondex SAU; Lleida, Spain). On arrival, birds were wing-banded, individually weighed and randomly allocated to cages (16 birds per cage) and assigned to one of the five dietary treatments (six replicas per treatment). Birds were housed in metabolic cages, with a grid floor and excreta collection tray, located in an environmentally controlled room. Throughout the study, feed and water were offered ad libitum. The temperature, humidity, ventilation, and illumination were automatically controlled, as recommended by the specifications in the Ross 308 lineage management handbook [14]. The animals and housing facilities were inspected, at least twice a day (d).

### 2.2. Experimental Design and Diets

All birds were raised with a starter feed until d 22 and a grower-finisher feed from d 23 to d 35, both in mash form. The wheat- and soybean meal-based diet was formulated to meet or exceed FEDNA’s (Fundación Española para el Desarrollo de la Nutrición Animal) requirements [15] and to minimize basal fat levels, as shown in Table 1. Titanium dioxide (TiO_2_) was included (5 g/kg) as an inert marker for determining the digestibility of FA.

The experimental diets consisted in a basal diet supplemented with 6% of the different fat sources (Table 2). The S was included at 6% (S6) and increasing amounts of PFAD were added in replacement of S: S4-PA2 (4% of S and 2% of PFAD), S2-PA4 (2% of S and 4% of PFAD) and PA6 (PFAD at 6%). The P was included at 6% (P6) as a control treatment for PFAD. Thus, 5 different diets were compared that were replicated 6 times. The composition of the experimental diets is shown in Table 3. The basal diet was manufactured at Pinsos Molinet S.A., (Prats de Lluçanès, Barcelona, Spain) and the addition of the experimental fat sources or fat blends to manufacture the experimental diets was performed at Lindo Pet Global S.A. (Castellar del Vallès, Barcelona, Spain).

### 2.3. Controls and Sampling

Individual body weight (BW) and feed consumption by cage were measured at 11, 22 and 35 d of age to calculate the average daily gain (ADG), average daily feed intake (ADFI) and the feed conversion ratio (FCR) throughout the experiment. Mortality was recorded and weighed to correct these parameters.

Two digestibility balances were performed in young animals from 9 to 11 d and in older animals from 33 to 35 d. At 11 d of age, 12 birds per cage were killed by cervical dislocation, and the jejunum (from the distal-most point of insertion of the duodenal mesentery to the junction with Meckel’s diverticulum), and ileum (from the junction with Meckel’s diverticulum to a point 1 cm proximal to the ileocecal junction) were carefully excised. Then, both segments (jejunum and ileum) were divided into 2 equal portions, named as upper and lower. Thus, for each cage, samples were taken of the digestive content from the upper and lower jejunum and the upper and lower ileum. The samples from the 12 birds from each cage were then homogenized and pooled (*n* = 6 per type of sample and dietary treatment). A representative sample of excreta (free of contaminants, such as feed or feathers) was also taken from each cage. Samples were frozen at −20 °C, and lyophilized. Thus, 5 different digesta samples were taken: 4 intestinal segments and excreta. Samples of diets, digesta and excreta were ground to pass through a 0.5-mm sieve and kept at 4 °C until further analyses. At 35 d of age, 2 birds per cage were euthanized, and the same procedure described above was carried out for sampling. In addition, at 35 d the abdominal fat pad (from the proventriculus surrounding the gizzard down to the cloaca) of each bird was removed and weighed. Abdominal fat pad weights were expressed in absolute values and as a percentage of BW.

### 2.4. Chemical Analysis

Oil samples were analyzed in triplicate for moisture and volatile matter according to the AOCS official method Ca 2d-25 [16], insoluble impurities [17], unsaponifiable matter according to the AOCS official method Ca 6b-53 [18], lipid-class composition according to IUPAC (2508 method) [19], and total FA composition [20], that were adapted to acid oils by Varona et al. [4]. The chemical analyses of the experimental fats are shown in Table 2.

Analytical determinations of the diets were performed according to the methods of AOAC International [21]: dry matter (Method 934.01), ash (Method 942.05), crude protein (Method 968.06), crude fiber (Method 962.09), and ether extract (EE) by Soxhlet analysis (Method 920.39). Gross energy was determined by an adiabatic bomb calorimeter (IKA C-4000, Janke-Kunkel; Staufen, Germany).

TiO_2_ in feed, digestive content and excreta was analyzed following the procedures of Short et al. [22] and determined by spectrophotometry ICP-OES (Optima 3200 RL, Perkin Elmer, Waltham, MA, USA).

The FA content of the feed, digestive content, and excreta was determined according to the method of Sukhija and Palmquist [23]. A direct extraction-transesterification procedure using nonadecanoic acid (C19:0; Sigma Aldrich Chemical Co.; St. Louis, MO, USA) as internal standard was performed. Then, the lipid extract was injected in a gas chromatograph (HP6890, Agilent Technologies; Waldbronn, Germany) under the conditions of the method previously described by Cortinas et al. [24]. FAs were identified based on the retention times of commercial standards of major FA (Supelco 37 component FAME Mix; Sigma-Aldrich Co.). Quantification was carried out by internal normalization. The macronutrient and FA compositions of the experimental diets are presented in Table 3.

The lipid-class composition (TAG, DAG, MAG, and FFA) of the feed, digestive content, and excreta was determined according to the IUPAC, 2508 method [19] by size-exclusion chromatography on an Agilent 1100 series HPLC chromatograph equipped with an isocratic pump, with the oven and a Refractive Index Detector (RID) both set at 35 °C (Agilent Technologies, Santa Clara, USA). Lipid extraction was previously performed following the methodology described by Rodriguez-Sanchez et al. [8] with slight modifications. Briefly, 0.1 g of lyophilized sample was weighed to extract the fat content with diethyl ether after acidification with HCl 1N. After lipid extraction, lipids were dissolved in 2 mL of tetrahydrofuran and filtered through a Nylon filter (13 mm, 0.45 μm), then 100 μL were injected (20 μL loop) into the HPLC. Separation was conducted using 2 Styragel columns (Styragel HR 1 and Styragel HR 0.5) of 30 cm × 0.78 cm i.d., filled with a spherical styrenedivinylbenzene copolymer of 5-μm particle size and pore sizes of 100 Å and 50 Å, respectively (Water Associates; Milford, MA, USA), connected in series. The mobile phase consisted of tetrahydrofuran (HPLC quality grade) at 1 mL/min. Lipid classes were identified by using standards for each lipid-class (trioleoylglycerol for TAG, dioleoylglycerol for DAG, oleoylglycerol for MAG and oleic acid for FFA; Sigma-Aldrich GmbH; Steinheim, Germany) and they were quantified according to their calibration curves.

### 2.5. Calculations

The apparent digestibility coefficients (ADC) of FA in each intestinal segment and the excreta were calculated according to the following formula using the TiO_2_ marker ratio in the diet and digesta or excreta.

ADC of FA = 1 − {(TiO_2_/FA)_d_/(TiO_2/_FA)_e_}, (1)

where (TiO_2_/FA)_d_ is the concentration of the inert marker and the FA in the diet (g/g DM), and (TiO_2_/FA)_e_ is the concentration of the inert marker and the FA in the digestive content or excreta (g/g DM).

The apparent metabolizable energy (AME) was calculated with the following equation:

AME (kcal/kg) = Apparent digestibility coefficient of gross energy (%) ∗ gross energy of the diet(2)

To determine the lipid-class content in the different GIT segments and excreta, the content of each lipid class present in the digestive tract of the chickens was estimated according to the following formula [12]:

Lipid-class content = [LC]/[TiO_2_], (3)

where [LC] is the concentration of the lipid-class in the digesta of the GIT segment or excreta (mg/g DM) and [TiO_2_] is the concentration of TiO_2_ in the digesta of the GIT segment or excreta (mg/g DM).

### 2.6. Statistical Analysis

The study design included 2 main factors: diet (5 treatments) × intestinal segment (5 types, being 4 intestinal segments and the excreta). The effect of the age was also compared as described below (11 vs. 35 d). The normality of the data and homogeneity of variance were verified. For each age, the effect of the diet on productive parameters (including abdominal fat depot at 35 d) and AME were statistically analyzed by one-way ANOVA using the GLM procedure of SAS (version 9.4, SAS Institute Inc., Cary, NC, USA) (*n* = 30; 5 diets × 6 replicas). For each age, and for each intestinal segment and excreta, the effect of the diet on the lipid-class content, FA digestibility, and its contribution on FA absorption was also evaluated by one-way ANOVA (*n* = 30; 5 diets × 6 replicas).

For each age, the effect of the intestinal tract on the lipid-class content was also analyzed by one-way ANOVA with the intestinal segments and the excreta as the main factor (*n* = 150; 30 samples × 5 types of digesta samples).

On each intestinal segment, the effect of the age (11 or 35 d) on FA absorption was statistically analyzed by one-way ANOVA using the age as the main factor (*n* = 60; 5 dietary treatments replicated 6 times × 2 ages). Additionally, for each dietary treatment, one-way ANOVA was used to test the effect of the age on feed AME, and at lower ileum level on lipid-class content and FA digestibility, (*n* = 12; 6 replicas of lower ileum × 2 ages).

The differences between treatments means were tested using Tukey’s correction for multiple comparisons. The cage served as the experimental unit, so there were six units per diet.

The results shown in tables are reported as least-square means, and in all statistical analyses, differences were considered significant at *p* < 0.05.

## 3. Results

### 3.1. Characterization of Experimental Oils and Diets

The characterization of the experimental oils included in the diets is shown in Table 2. The main FAs in S were linoleic and oleic acids, whereas in P and PFAD they were palmitic and oleic acids. The unsaturated-to-saturated FA ratio (UFA:SFA) was higher for S (5.29) than for PFAD and P (0.82 and 0.98, respectively). Regarding lipid-class composition, S and P were mainly composed of TAG (>92%), whereas PFAD was mainly composed of FFA (92.94%). The rest of parameters observed for PFAD were in line with those usually found in PFAD [4], being insoluble impurities and unsaponifiable matter higher in PFAD than in P.

The chemical analysis of the experimental diets is shown in Table 3. Replacing S with PFAD led to an increment in both saturation degree and FFA content. Therefore, a progressive decrease in the UFA:SFA from 4.16 to 1.14 in starter diets, and from 4.54 to 1.15 in grower-finisher diets was obtained. In parallel, a large increase was achieved in the FFA content from 14.20% to 79.17% in starter diets and from 10.61% to 78.44% in grower-finisher diets. Although the FA profile and saturation degree of P6 and PA6 were similar, their FFA content was different (P6: 8–9% FFA; PA6: 78–79% FFA).

### 3.2. Growth Performance and Abdominal Fat Deposition

The effect of dietary fat source on growth-performance traits in starter (from 0 to 22 d), grower-finisher (from 23 to 35 d) and the global (from 0 to 35 d) periods, and on abdominal fat deposition is reported in Table 4. No significant differences in any of the performance parameters, nor any feeding period (*p* > 0.05), were observed due to the saturation degree or FFA content of the diet. Concerning the effect of the diet on abdominal fat deposition, a tendency for a reduction of fat weight (%) was observed as S was replaced by PFAD (*p* = 0.08).

### 3.3. Lipid-Class Content along the Intestinal Tract

The lipid-class content (TAG, DAG, MAG, and FFA) in the upper and lower jejunum, upper and lower ileum and excreta determined in 11 and 35-d-old broiler chickens fed the different experimental diets is shown in Table 5 and Table 6, respectively. In general, a significant decrease in TAG, DAG, and FFA content was observed from the upper jejunum to lower ileum (*p* < 0.001) (Supplementary Table S1).

Significant differences in TAG, DAG and MAG content in the different diets were obtained at the jejunum level in starter broiler chickens (Table 5; *p* ≤ 0.027). In contrast, grower broiler chickens showed no differences among the different dietary treatments for TAG, DAG, and MAG content in any intestinal segment (Table 6). For each experimental diet, TAG and DAG content at the lower ileum level was significantly lower in grower chickens than in starter chicks (*p* ≤ 0.02) (Supplementary Table S2).

Regardless of the diet, FFA was the major lipid-class present in the digesta from the upper jejunum to the excreta, both in starter and grower chickens. In starter broiler chickens, FFA content decreased notably from the upper to lower jejunum (*p* < 0.001), while in grower broiler chickens, the significant decrease reached the upper ileum (*p* < 0.001) (Supplementary Table S1).

**Figure 1:**
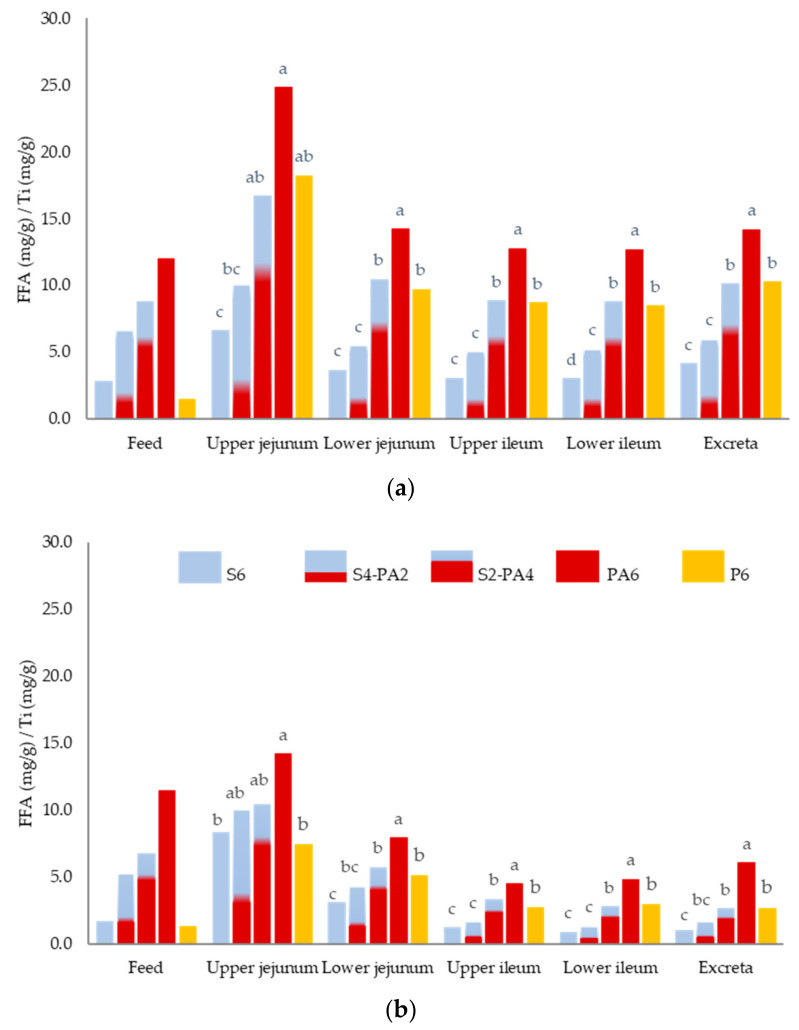
FFA content ^1^ in the feed, upper jejunum, lower jejunum, upper ileum, lower ileum, and excreta for the five different diets; with 6% of soybean oil (S6), blend with 4% soybean oil and 2% palm fatty acid distillate (S4-2PA), blend with 2% soybean oil and 4% palm fatty acid distillate (S2-2PA), with 6% of palm fatty acid distillate (PA6) and with 6% palm oil (P6) in (**a**) 11-d-old broiler chickens and (**b**) 35-d-old broiler chickens. ^1^ FFA concentration (mg/g)/Ti concentration (mg/g) in each intestinal segment and excreta. Values are pooled means of 6 replicates per each diet with 12 chickens/replicate at 11 d, and 2 chickens/replicate at 35 d. a–d: columns not sharing a common letter within each intestinal segment are significantly different (*p* ≤ 0.01).

Differences in FFA content were also observed among the dietary treatments in the digesta in all intestinal segments and in the excreta, at 11 d (*p* < 0.001) and 35 d (*p* ≤ 0.011) (Table 5; Table 6, Figure 1). It was found that the higher the replacement of S by PFAD in the diet, the higher the FFA content in the digesta, which was more evident from the lower jejunum on.

In both starter and grower broiler chickens, birds fed the most unsaturated diets (S6 and S4-PA2) showed the lowest FFA values in the digesta of most of the GIT segments studied and the excreta (*p* > 0.001). No differences were observed in the FFA content of the digesta between chickens fed the S2-PA4 and P6 diets along the intestinal tract and excreta. Chickens fed PA6 had the highest FFA content. For each experimental diet, it was observed that starter chicks had a higher FFA content at the lower ileum level than grower chickens (*p* < 0.001) (Supplementary Table S2).

### 3.4. Apparent Fatty-Acid Digestibility along the Intestinal Tract

Table 7 and Table 8 show the feed apparent metabolizable energy and apparent FA digestibility coefficients in the different intestinal segments and excreta determined in 11 and 35-d-old broiler chickens fed the different dietary treatments, respectively.

Differences were observed in the feed AME values among the different diets (*p* < 0.001) both in 11-d-old broiler chickens (Table 7) and in 35-d-old broiler chickens (Table 8). In general, at both ages, the lowest values were obtained in the PA6 diet and the highest in the most unsaturated diets (S6 and S4-PA2). An increase in AME values was observed in grower chickens compared to starter chicks fed diets with higher SFA and FFA contents (PA6, S2-PA4, and P6; *p* ≤ 0.002) (Supplementary Table S3).

Starter broiler chicks fed the diets containing PA6 showed the lowest digestibility coefficients, mainly for TFA and SFA from the lower jejunum on (*p* < 0.001) (Table 7). For S6 and S4-PA2, no differences were obtained in TFA, MUFA or PUFA along the GIT or in the excreta, and these diets showed the highest TFA digestibility coefficient values. In contrast, birds fed the S2-PA4 diet had lower SFA digestibility coefficients than those fed the S6 diet from the lower jejunum on (*p* < 0.001). Comparing S2-PA4 and P6, no differences were obtained in either the TFA or all FA group digestibility coefficients in the GIT segments and excreta examined (except for PUFA at the lower jejunum).

In grower chickens (35 d), birds fed the PA6 diet showed the lowest digestibility coefficients for TFA and SFA only in the lower ileum and excreta (*p* < 0.001) and the lowest MUFA digestibility coefficient in the excreta (*p* < 0.001) (Table 8). No differences were observed between S6 and S4-PA2 in TFA and all FA group digestibility coefficients. The highest TFA, SFA, and PUFA digestibility coefficients were shown from the lower jejunum on. Comparing S2-PA4 and P6, no significant differences were observed in TFA or SFA digestibility (except for SFA in the lower ileum). In contrast, birds fed the S2-PA4 diet had lower MUFA digestibility coefficients than those fed P6 diet from the lower jejunum on (*p* < 0.001), and higher PUFA digestibility coefficients in the upper ileum and excreta (*p* < 0.001).

For each experimental diet, chickens at d 35 had higher FA digestibility coefficients at the lower ileum level than chicks at d 11 (*p* ≤ 0.05) (Supplementary Table S3).

### 3.5. Contribution of Each Intestinal Segment to FA Absorption

**Figure 2:**
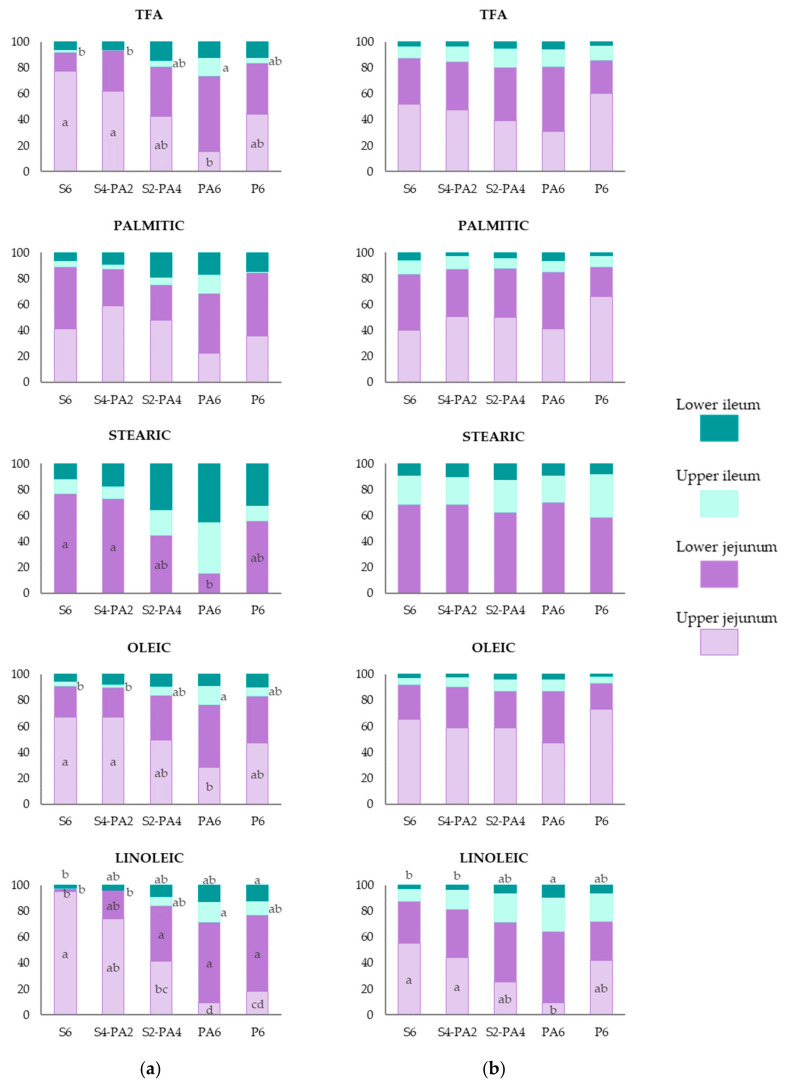
Contribution of each intestinal segment to the apparent fatty acid digestibility, calculated as a proportion of total digestibility reached at the lower ileum, along the intestinal tract for the five different diets; with 6% of soybean oil (S6), blend with 4% soybean oil and 2% palm fatty acid distillate (S4-2PA), blend with 2% soybean oil and 4% palm fatty acid distillate (S2-2PA), with 6% of palm fatty acid distillate (PA6) and with 6% palm oil (P6) in (**a**) 11-d-old broiler chickens and (**b**) 35-d-old broiler chickens. TFA (Total Fatty Acids), Palmitic (C16:0), stearic (C18:0), oleic (C18:1n-9) and linoleic (C18:2n-6) acids. Values are means of 6 replicates per each diet with 12 chickens/replicate at 11 d, and 2 chickens/replicate at 35 d. a–d: columns with the same intestinal segment not sharing a common letter are significantly different (*p* < 0.01).

To better understand the importance of the different intestinal segments in the FA absorption, the contribution of each intestinal segment was calculated as a proportion of the total digestibility coefficient obtained in the lower ileum, since it is well known that this is the last segment where absorption can take place [25]. The contributions of the different intestinal segments to the digestibility of TFA and the four major FAs (palmitic and stearic, representing SFA; oleic, MUFA; and linoleic, PUFA) are shown in Figure 2.

The results show that jejunum was the main site of TFA absorption (Jejunum: 84%; Ileum: 16%; these results indicate the percentage of FA disappearance), when all diets at 11 d and 35 d are considered. It was also the most important place for the absorption of palmitic (11 d: 80%; 35 d: 87%), oleic (11 d: 85%; 35 d: 89%), and linoleic acids (11 d: 85%; 35 d: 75%).

In starter broiler chickens (Figure 2a), the contribution of the upper jejunum to the absorption of TFA decreased as S was replaced by PFAD (S6: 77%a, S4-PA2: 62%a, S2-PA4: 42%ab, PA6: 15%b, P6: 44%ab; *p* = 0.002) inversely to the increased contribution of the following segments, mainly the lower jejunum. A similar pattern was observed for the absorption of oleic and linoleic acids. In relation to SFA, no differences among diets in the contribution of the jejunum (both upper and lower) were observed for palmitic acid. For stearic acid the absorption started in the lower jejunum and was higher for chicks fed S6 (77%) and S4-PA2 (72%) than for those fed the PA6 (15%) diet (*p* = 0.004). In parallel, the contribution of the ileum (upper and lower segments) to the absorption of stearic acid increased as more S was replaced by PFAD (higher saturation and higher FFA content), reaching 85% in the PA6 diet.

In grower broiler chickens, no differences among diets in the contribution of the different intestinal segments to FA absorption were observed, except for linoleic acid. The absorption of linoleic acid in the upper jejunum was 55% and 44% for S6 and S4-PA2, respectively, and 9% for PA6 (Figure 2b; *p* = 0.003). Conversely, in the lower ileum, 9% of linoleic acid was absorbed for chickens fed PA6, compared to 3% for chickens fed the S6 and S4-PA2 diets (*p* = 0.010). As observed in starter chicks, the absorption of stearic acid is delayed, starting at the lower jejunum level, but no effect of the degree of saturation and FFA content of the diet was obtained. The upper and lower ileum make a large contribution to the absorption of stearic acid (25% and 10% on average, respectively).

The TFA absorption was higher in the upper ileum for grower chickens (*p* < 0.001) and in the lower ileum for starter chicks (*p* < 0.001) (Supplementary Table S4).

## 4. Discussion

Studying the lipid classes and FA digestibility along the intestinal tract leads to a better understanding of the dynamics of the hydrolysis-absorption process of PFAD alone and in blends in broiler chickens. Our results show that lipolysis, based on the disappearance of TAG and DAG in the digesta, is extended until the ileum. In addition, the results obtained support that hydrolysis is not the most limiting step for fat utilization when compared with the absorption process, which is in accordance with our previous studies in vitro [26] and in vivo [12]. Furthermore, our results suggest that hydrolysis efficiency is mainly affected by the age of the bird, whereas the lipid composition of the diet (saturation and FFA level) has less influence on this process. An improvement in the hydrolysis capacity with chicken age was demonstrated by the higher disappearance of TAG and DAG at 35 d compared to 11 d. In starter broiler chickens, some limitations in the hydrolysis process due to low bile and lipase secretion have been described [27]. However, Noy and Sklan [28] reported an increase of 80% in the duodenal bile acid secretion between 10 d and 21 d, and a 20-fold increase in lipase secretion between 4 d and 21 d.

The absorption process takes place as a dynamic process parallel to the hydrolysis of fat. The content of the end lipolysis products, mainly FFA, decreased from the upper jejunum to ileum and the maximum digestibility coefficients of FA were reached in the lower ileum. These results show that the lower ileum is the last segment where FA absorption occurs. Moreover, the evolution of the FFA content and the digestibility values throughout the gut confirmed that the jejunum was the main site of FA absorption, in line with previous studies on broiler chickens [7,12]. However, the absorption dynamics along the GIT are different according to the FA, and the stearic acid is the one that is absorbed later with no absorption observed until the lower jejunum at both ages. This is related to the lower solubilization into DMM for long-chain saturated FA compared to unsaturated long-chain FA [29]. This is also reflected in the lower digestibility coefficients for SFA along the GIT compared to MUFA or PUFA, regardless of the age of the chicken or the diet.

The results on the effect of the diet provide evidence of the detriment to the dietary AME values, FA digestibility coefficients, especially SFA, and FFA absorption associated with both the higher SFA% and FFA% of the broiler chicken diet. The lower FA absorption together with the higher residual FFA content in the digesta at the lower ileum obtained for chickens fed the higher level of PFAD (6%; PA6), could be explained by two factors. First, the association of FFA, mainly SFA, with minerals to form insoluble soaps has been described, so that both the FFA and the mineral become unavailable for the absorption [30]. This has a greater impact on young birds than on older ones [31]. In our last in vitro study [26], we found that fat content from PFAD compared to other fat sources (P, S, or soybean acid oil) was less available for micellar solubilization. Second, and related to the lipid-class content, the lowest MAG content in PA6 diets (Table 3; 1.5% on average for both ages) may hinder the absorption of many FFAs [9] since the emulsifying properties of MAG improve the rate of FA incorporation into DMM [32]. This in turn could explain that birds fed PA6 tended to show the lowest abdominal fat weight (%).

The potential inclusion of PFAD in feed for broiler chickens is influenced by the age of the bird. In 11-d-old broiler chicks, the supplementation of PFAD at any level studied had a negative effect on fat utilization compared to S. Consistent with our results, several authors (Wiseman and Salvador, [33]; Vilarrasa et al. [5]; Rodriguez-Sanchez et al. [12]) have found a negative impact of dietary saturation and FFA level on fat utilization in broiler chickens. At 35 d the PA6 showed the worst fat utilization, however, adding PFAD in substitution of S with a feed FFA content up to 30% and a UFA:SFA ratio higher than 2.6 made it possible to achieve a high level of fat digestibility, similar to that obtained using S. This could be partially related to the higher FA digestibility coefficients and higher dietary AME values obtained for the S4-PA2 diet compared to those calculated from the proportions of the components. This suggests a positive synergic effect by the presence of UFA together with the presence of different lipid-class structures provided by S, since UFA and MAG obtained from the lipolysis of TAG are natural emulsifiers, which might enhance the incorporation of SFA, mainly FFA of PFAD, in the DMM and increase its absorption [34]. This synergistic effect is in agreement with the reported positive results of blending saturated and unsaturated conventional lipid sources [11] and acid oils [10]. However, the similarities obtained in feed AME values, lipid-class content in digesta, and apparent FA digestibility coefficients for S2-PA4 (UFA: SFA ratio: 1.7; FFA%: 53) and P6 (UFA: SFA ratio: 1.3; FFA%: 9) suggest that changes in the saturation degree might have a greater impact on FA utilization than the changes in the FFA level of the diet, as reported by Vilarrasa et al. [5] and Rodriguez-Sanchez et al. [12].

The present results also demonstrated that replacing S with PFAD led to a delay in the FA absorption along the GIT, which was more evident in starter animals (11-d-old chicks), and for the absorption of linoleic acid in 35-d-old chickens. Thus, even though the jejunum is the main site of fat absorption, the differentiation between the upper and the lower segments should be considered for future studies, at least in starter broiler chickens.

The comparison of the results between starter (11 d) and grower (35 d) broiler chickens confirms that the age has a positive effect on the FFA lipid-class absorption, FA absorption, and AME values of all the diets, which is consistent with the findings of Batal and Parsons [35], Tancharoenrat et al. [11], Roll et al. [13], Rodriguez-Sanchez et al. [8], and Viñado et al. [6]. However, it is important to highlight that the observed improvement with age in FA digestibility (especially for SFA), FFA absorption, and dietary AME values, was higher for those chickens fed the most saturated diets (especially with higher FFA%) than for those fed the most unsaturated diets. That there were no differences among diets in grower chickens in the contribution of intestinal segments to FA absorption suggests that the absorption of diets with higher SFA% and FFA% is advanced to the upper intestinal segments at 35 d. This was especially evident for the absorption of stearic acid, and the contribution of the lower jejunum increased due to the absorption of this FA acid in grower broiler chickens fed the PA6 diet. In addition, the higher contribution of the upper ileum in TFA and linoleic acid absorption at 35 d suggests that this segment plays a key role in improving FA absorption with age. In starter chickens, the limited capacity of fat absorption [27] together with the shorter feed retention time in the GIT (3.15 h in 11-d-old chicks and 5.10 h in 42-d-old chickens) [36] could explain the lower efficiency in the absorption process. This in turn could explain the higher implication of the lower ileum in young chicks as it is the last part of the GIT for the remaining FA to be absorbed. Therefore, it may be recommendable to separate the ileum into upper and lower segments for further studies. Determining the maximum fat utilization at the lower ileum level instead of from the pool of digesta of the whole ileum may give more accurate results.

The maximum digestibility coefficients of SFA reached at the lower ileum, show that at 11 d both the dietary FFA content (PA6: 0.18 vs. P6: 0.48) and the SFA level (P6: 0.48 vs. S6: 0.69) had a great impact. At 35 d the magnitude of the negative effect was lower than at 11 d, for the dietary FFA content (PA6: 0.64 vs. P6: 0.78) and for the SFA level (P6: 0.78 vs. S6: 0.90). This in turn suggests that as age increases, the digestibility of SFA improves and, most notably, the utilization of FFA improves.

## 5. Conclusions

The present study confirms that determining the lipid classes together with the FA digestibility along the GIT provides valuable information for better understanding the dynamics of FA utilization in diets with different FA profiles and FFA contents. The results demonstrate that the effect of dietary saturation degree (UFA:SFA) on dietary fat utilization is higher than the effect of dietary FFA level. A clear improvement in the efficiency of both the lipolysis and the absorption process was observed with age. Fat hydrolysis is more affected by the age of the chicken than by the saturation degree and/or FFA content of the diet. The absorption results demonstrated that most of the FA absorption occurs in the jejunum (from 73% to 92%), but the ileum also plays a key role (from 8% to 27%). The contribution of the upper and lower segments of the jejunum and ileum to FA absorption is influenced by the characteristics of the dietary FA (degree of saturation, chain length, and FFA%), and the age of the chicken. There is a notably higher implication of the upper ileum for grower broiler chickens.

Replacing soybean oil with palm fatty acid distillate affected the extent and the site of FA absorption. The results show that the increase in SFA% and FFA% in the diet reduced and delayed the absorption of the dietary FA, especially the SFA in starter broiler chicks. As age increased, the FA absorption increased, and advanced to the upper intestinal segments, especially in the most saturated and rich FFA diets. Age has a positive effect on the digestibility of SFA and, above all, on the FFA utilization. For 11-day-old starter broiler chickens, it is not recommended to use this by-product alone or in blends. For grower broiler chickens, it is possible to include palm fatty acid distillate blended with a conventional unsaturated oil, such as soybean oil, in feed formulation, when the blend has from 2.6 UFA:SFA and the FFA% does not exceed 30%, without impairing FA utilization or growth performance. This potential strategy for using palm fatty acid distillate without negatively impacting fat utilization by the animal implies a reduction in costs and a way to upcycle and valorize this by-product.

## Figures and Tables

**Table 1:** Ingredient composition of the experimental basal diet.

Ingredients, %	Starter Diet (from 0 d to 22 d)	Grower-Finisher Diet (from 23 d to 35 d)
Wheat	54.49	44.02
Soybean meal 47%	35.40	27.25
Barley	-	18.58
Experimental fats ^1^	6.00	6.00
Calcium carbonate	1.44	1.39
Monocalcium phosphate	0.99	1.20
Titanium dioxide	0.50	0.50
Vitamin and mineral premix ^2^	0.40	0.40
Sodium chloride	0.40	0.35
DL-Methionine	0.23	0.17
L-Lysine	0.15	0.12
L-Threonine	-	0.02

^1^ Soybean oil, palm oil and palm fatty acid distillate in different proportions. ^2^ Provides per kg of feed: vitamin A (from retinol), 10,000 IU; vitamin D3 (from cholecalciferol), 4800 IU; vitamin E (from alfa tocopherol), 45 mg; vitamin B1, 3 mg; vitamin B2, 9 mg; vitamin B6, 4.5 mg; vitamin B12, 40 µg; vitamin K3, 3 mg; calcium pantothenate, 16.5 mg; nicotinic acid, 51 mg; folic acid, 1.8 mg; biotin, 150 µg; Fe (from FeSO_4_·7H_2_O), 54 mg; I (from Ca(I_2_O_3_)_2_), 1.2 mg; Cu (from CuSO_4_·5H_2_O), 12 mg; Mn (from MnO), 90 mg; Zn (from ZnO), 66 mg; Se (from Na_2_SeO_3_), 0.18 mg; β-glucanase 150 U; xylanase 270 U.

**Table 2:** Chemical analyses of the experimental fats ^1^.

Item	S	PFAD	P
Moisture (g/100 g)	ND	0.01	ND
Insoluble impurities (g/100 g)	1.27	3.76	0.59
Unsaponifiable matter (g/100 g)	0.99	1.34	0.21
Fatty acid composition (%) ^2^			
C16:0	10.98	46.59	43.94
C18:0	3.47	6.62	4.64
C18:1 n-9	25.11	34.96	38.43
C18:2 n-6	51.70	8.49	9.70
C18:3 n-3	5.34	0.29	0.13
Minor fatty acids	3.40	3.05	3.15
SFA	15.86	55.13	50.64
*cis-*MUFA	27.06	35.87	39.44
*trans-*C18:1	0.04	0.22	0.08
PUFA	57.04	8.78	9.83
UFA:SFA	5.29	0.82	0.98
Lipid class composition (%) ^3^			
TAG	96.27	4.01	92.46
DAG	3.23	3.04	7.54
MAG	ND	ND	ND
FFA	0.50	92.94	ND
T (mg/kg)	1007.31	42.79	199.40
T3 (mg/kg)	ND	52.59	431.87

Abbreviations: S, soybean oil; P, palm oil; PFAD, palm fatty acid distillate; SFA, saturated fatty acids; MUFA, monounsaturated fatty acids; PUFA, polyunsaturated fatty acids; UFA:SFA, unsaturated to saturated fatty acid ratio, calculated as described by Varona et al. [4]; TAG, triacylglycerols; DAG, diacylglycerols; MAG, monoacylglycerols; FFA, free fatty acids; T, sum of α-, β-, γ- and δ-tocopherols; T3, sum of α-, β-, γ- and δ-tocotrienols; ND, not detected. ^1^ Chemical composition analyzed as described by Varona et al. [4]. ^2^ Percentage of total fatty acids (normalized data, area %); ^3^ Percentage of total lipid classes (normalized data, area %).

**Table 3:** Analyzed ^1^ macronutrient content and fatty-acid and lipid-class composition of the experimental diets ^2^.

Item	Starter Diets (from 0 to 22 d)					Grower-Finisher Diets (from 23 to 35 d)				
	S6	S4-PA2	S2-PA4	PA6	P6	S6	S4-PA2	S2-PA4	PA6	P6
Macronutrient content										
Dry matter (g/100 g)	91.00	91.03	91.14	90.89	90.93	90.14	90.27	90.37	90.43	90.02
Crude protein (g/100 g)	23.61	23.87	23.47	23.60	23.15	21.04	22.03	21.45	20.59	20.84
Crude fat (g/100 g)	7.51	7.39	8.16	7.78	7.70	8.18	8.08	8.36	8.10	7.49
Crude fiber (g/100 g)	3.29	3.14	3.14	2.86	3.20	3.08	3.10	3.32	3.13	3.41
Ash (g/100 g)	5.54	5.58	6.92	7.13	7.09	6.21	6.69	6.51	6.46	5.75
Gross energy, kcal/kg	4367	4402	4368	4332	4332	4339	4355	4320	4308	4324
Fatty acid composition (%)										
C14:0	-	0.40	0.66	0.91	0.87	0.06	0.39	0.65	0.90	0.85
C16:0	14.43	21.99	30.79	39.07	37.38	13.24	21.93	30.24	39.18	36.85
C18:0	3.48	4.19	5.07	5.79	4.29	3.35	4.19	4.97	5.69	4.17
C18:1 n-9	22.83	25.07	27.57	30.31	32.34	22.61	25.31	27.85	29.96	32.49
C18:1 n-7	1.46	1.28	1.05	0.78	0.83	1.50	1.25	1.00	0.74	0.80
C18:2 n-6	50.78	41.48	30.59	20.30	21.37	52.04	41.29	30.99	20.72	22.02
C18:3 n-3	5.27	4.13	2.83	1.55	1.46	5.50	4.20	2.93	1.61	1.60
Minor fatty acids	1.75	1.46	1.45	1.29	1.46	1.69	1.44	1.38	1.19	1.22
SFA	18.72	27.26	37.14	46.14	43.13	17.70	27.20	36.38	46.07	42.47
MUFA	25.24	27.13	29.44	32.01	34.04	24.76	27.31	29.70	31.60	33.92
PUFA	56.04	45.61	33.42	21.85	22.83	57.54	45.49	33.92	22.33	23.62
UFA:SFA	4.16	2.60	1.66	1.14	1.30	4.54	2.61	1.70	1.15	1.34
Lipid class composition (%)										
TAG	71.88	54.76	37.54	14.98	78.67	76.67	58.78	37.77	14.20	78.53
DAG	11.73	10.05	7.47	4.67	10.54	10.23	9.03	7.28	5.58	10.44
MAG	2.19	2.10	1.66	1.19	1.84	2.49	2.20	1.93	1.77	2.31
FFA	14.20	33.08	53.33	79.17	8.96	10.61	29.99	53.01	78.44	8.72

Abbreviations: SFA, saturated fatty acids; MUFA, monounsaturated fatty acids; PUFA, polyunsaturated fatty acids; UFA:SFA, unsaturated to saturated fatty acid ratio, calculated as described by Varona et al. [4]; TAG, triacylglycerols; DAG, diacylglycerols; MAG, monoacylglycerols; FFA, free fatty acids. ^1^ All samples were analyzed at least in duplicate. ^2^ Dietary treatments supplemented with 6% of soybean oil (S6), palm fatty acid distillate (PA6), palm oil (P6), or oil blends with 4% soybean oil and 2% palm fatty acid distillate (S4-PA2) or 2% soybean oil and 4% palm fatty acid distillate (S2-PA4). In all cases, fatty acids and lipid classes are expressed as internal area normalization (in %).

**Table 4:** Growth performance and abdominal fat pad deposition of broiler chickens according to different fat sources in diet ^1^.

Item	Dietary Treatments ^2^					SEM ^3^	*p*-Value
	S6	S4-PA2	S2-PA4	PA6	P6		
From 0 to 22 d							
ADFI, g/d/bird	48.7	53.2	50.7	54.0	54.5	2.25	0.335
ADG, g/d/bird	37.2	39.1	38.6	39.7	40.6	1.24	0.373
FCR, g/g	1.31	1.36	1.31	1.36	1.34	0.036	0.733
BW at 22 d, g	856	899	888	913	933	27.1	0.361
From 23 to 35 d							
ADFI, g/d/bird	134	141	141	144	143	2.92	0.148
ADG, g/d/bird	87.8	90.2	89.8	90.3	89.9	2.18	0.929
FCR, g/g	1.53	1.57	1.57	1.59	1.60	0.022	0.175
BW at 35 d, g	1997	2072	2055	2086	2101	44.4	0.526
From 0 to 35 d							
ADFI, g/d/bird	80.3	85.8	84.3	87.3	87.5	2.01	0.130
ADG, g/d/bird	56.0	58.1	57.6	58.5	58.9	1.27	0.535
FCR, g/g	1.43	1.48	1.46	1.49	1.49	0.017	0.154
Abdominal fat, g	29.62	30.35	29.52	25.66	32.36	1.938	0.136
Abdominal fat, %	1.46	1.47	1.42	1.23	1.53	0.082	0.080

Abbreviations: ADFI, average daily feed intake; ADG, average daily gain; FCR, feed conversion ratio; BW, body weight. ^1^ Diets supplemented with 6% of soybean oil (S6), palm fatty acid distillate (PA6), palm oil (P6), or oil blends with 4% soybean oil and 2% palm fatty acid distillate (S4-PA2) or 2% soybean oil and 4% palm fatty acid distillate (S2-PA4). ^2^ Values are pooled means of 6 replicates with 16 chickens/replicate from 0 to 11 d and 4 chickens/replicate from 11 to 35 d. In the case of BW, values are means of 24 chickens each treatment from 22 to 35 d. For abdominal fat, values are means of 2 chickens/replicate: 12 for each treatment at 35 d. ^3^ SEM, standard error of means of 6 observations per treatment (the experimental unit is the cage).

**Table 5:** Lipid-class content ^1^ along the intestinal tract and excreta according to different fat sources in the diet ^2^ in 11-d-old broiler chickens.

Item	Dietary Treatments					SEM ^3^	*p*-Value
	S6	S4-PA2	S2-PA4	PA6	P6		
Upper Jejunum							
TAG	0.53 ^ab^	0.57 ^a^	0.44 ^ab^	0.19 ^b^	0.49 ^ab^	0.082	0.027
DAG	1.30	1.60	1.91	2.37	1.99	0.413	0.440
MAG	0.18 ^ab^	0.28 ^a^	0.16 ^ab^	0.12 ^b^	0.15 ^ab^	0.033	0.018
FFA	6.58 ^c^	10.04 ^bc^	16.69 ^ab^	24.82 ^a^	18.20 ^ab^	2.215	<0.001
Lower Jejunum							
TAG	0.34 ^ab^	0.34 ^ab^	0.47 ^a^	0.32 ^b^	0.30 ^b^	0.031	0.008
DAG	0.82 ^ab^	0.69 ^b^	1.16 ^a^	0.98 ^ab^	0.76 ^b^	0.091	0.008
MAG	0.18	0.18	0.21	0.17	0.17	0.025	0.729
FFA	3.65 ^c^	5.50 ^c^	10.45 ^b^	14.21 ^a^	9.70 ^b^	0.471	<0.001
Upper Ileum							
TAG	0.25	0.24	0.25	0.32	0.24	0.053	0.775
DAG	0.64	0.55	0.72	0.87	0.62	0.121	0.426
MAG	0.16	0.15	0.15	0.16	0.13	0.030	0.929
FFA	3.05 ^c^	5.02 ^c^	8.82 ^b^	12.76 ^a^	8.69 ^b^	0.530	<0.001
Lower Ileum							
TAG	0.32	0.29	0.27	0.33	0.19	0.045	0.239
DAG	0.64	0.60	0.76	0.71	0.45	0.084	0.114
MAG	0.23	0.25	0.25	0.23	0.15	0.029	0.137
FFA	3.02 ^d^	5.23 ^c^	8.78 ^b^	12.70 ^a^	8.47 ^b^	0.471	<0.001
Excreta							
TAG	0.38	0.39	0.28	0.27	0.29	0.044	0.219
DAG	0.98	0.95	1.33	1.20	0.80	0.167	0.205
MAG	0.19	0.13	0.15	0.13	0.21	0.031	0.282
FFA	4.16 ^c^	5.98 ^c^	10.11 ^b^	14.18 ^a^	10.24 ^b^	0.794	<0.001

Abbreviations: TAG, triacylglycerols; DAG, diacylglycerols; MAG, monoacylglycerols; FFA, free fatty acids. ^1^ Lipid-class concentration (mg/g)/Ti concentration (mg/g) in each intestinal segment and excreta. ^2^ Values are pooled means of 6 replicates with 12 chickens/replicate fed diets supplemented with 6% of soybean oil (S6), palm fatty acid distillate (PA6), palm oil (P6), or oil blends with 4% soybean oil and 2% palm fatty acid distillate (S4-PA2) or 2% soybean oil and 4% palm fatty acid distillate (S2-PA4). ^3^ SEM = standard error of the mean. a–d: means in a row not sharing a common letter are significantly different (*p* < 0.05).

**Table 6:** Lipid-class content ^1^ along the intestinal tract and excreta according to different fat sources in the diet ^2^ in 35-d-old broiler chickens.

Item	Dietary Treatments					SEM ^3^	*p-*Value
	S6	S4-PA2	S2-PA4	PA6	P6		
Upper Jejunum							
TAG	0.21	0.20	0.20	0.20	0.26	0.048	0.871
DAG	1.32	1.69	1.47	2.22	1.20	0.256	0.073
MAG	0.24	0.27	0.30	0.27	0.17	0.048	0.436
FFA	8.28 ^b^	10.05 ^ab^	10.40 ^ab^	14.18 ^a^	7.40 ^b^	1.296	0.011
Lower Jejunum							
TAG	0.10	0.26	0.27	0.23	0.22	0.045	0.108
DAG	0.43	0.61	0.61	0.72	0.58	0.115	0.496
MAG	0.18	0.26	0.19	0.23	0.14	0.041	0.342
FFA	3.10 ^c^	4.29 ^bc^	5.72 ^b^	7.89 ^a^	5.08 ^b^	0.412	<0.001
Upper Ileum							
TAG	0.14	0.16	0.13	0.13	0.09	0.021	0.254
DAG	0.16	0.21	0.18	0.17	0.17	0.021	0.482
MAG	0.11	0.12	0.16	0.14	0.11	0.015	0.090
FFA	1.20 ^c^	1.65 ^c^	3.32 ^b^	4.47 ^a^	2.67 ^b^	0.189	<0.001
Lower Ileum							
TAG	0.09	0.11	0.09	0.07	0.07	0.022	0.676
DAG	0.14	0.17	0.11	0.18	0.17	0.020	0.141
MAG	0.13	0.14	0.21	0.21	0.15	0.022	0.051
FFA	0.87 ^c^	1.32 ^c^	2.77 ^b^	4.82 ^a^	2.92 ^b^	0.257	<0.001
Excreta							
TAG	0.12	0.21	0.18	0.15	0.15	0.021	0.080
DAG	0.13	0.20	0.14	0.24	0.15	0.027	0.055
MAG	0.09 ^b^	0.12 ^ab^	0.12 ^ab^	0.16 ^a^	0.13 ^ab^	0.011	0.005
FFA	0.96 ^c^	1.63 ^bc^	2.62 ^b^	6.04 ^a^	2.66 ^b^	0.265	<0.001

Abbreviations: TAG, triacylglycerols; DAG, diacylglycerols; MAG, monoacylglycerols; FFA, free fatty acids. ^1^ Lipid-class concentration (mg/g)/Ti concentration (mg/g) in each intestinal segment and excreta. ^2^ Values are pooled means of 6 replicates with 2 chickens/replicate fed diets supplemented with 6% of soybean oil (S6), palm fatty acid distillate (PA6), palm oil (P6), or oil blends with 4% soybean oil and 2% palm fatty acid distillate (S4-PA2) or 2% soybean oil and 4% palm fatty acid distillate (S2-PA4). ^3^ SEM = standard error of the mean. a–c: means in a row not sharing a common letter are significantly different (*p* < 0.05).

**Table 7:** Feed apparent metabolizable energy values and apparent fatty-acid digestibility coefficients along the intestinal tract and excreta according to different fat sources in the diet in 11-d-old broiler chickens.

Item	Dietary Treatments ^1^					SEM ^4^	*p*-Value
	S6	S4-PA2	S2-PA4	PA6	P6		
AME, kcal/kg ^2^	3348 ^a^	3340 ^a^	3074 ^b^	2760 ^c^	3014 ^b^	26.08	<0.001
Upper Jejunum ^3^							
TFA	0.61 ^a^	0.61 ^a^	0.29 ^b^	0.05^b^	0.20 ^b^	0.071	<0.001
SFA	0.20 ^ab^	0.36 ^a^	0.19 ^ab^	-0.01^b^	0.19 ^ab^	0.076	0.044
MUFA	0.51 ^a^	0.51 ^a^	0.35 ^ab^	0.16 ^b^	0.31 ^ab^	0.059	<0.001
PUFA	0.78^a^	0.71 ^a^	0.34 ^b^	0.02 ^b^	0.04 ^b^	0.078	<0.001
Lower Jejunum ^3^							
TFA	0.72 ^a^	0.67 ^a^	0.51 ^b^	0.30 ^c^	0.48 ^b^	0.020	<0.001
SFA	0.60 ^a^	0.47 ^b^	0.32 ^c^	0.12 ^d^	0.37 ^c^	0.026	<0.001
MUFA	0.69 ^a^	0.67 ^ab^	0.58 ^b^	0.44 ^c^	0.58 ^b^	0.025	<0.001
PUFA	0.77 ^ab^	0.78 ^a^	0.67 ^b^	0.48 ^c^	0.56 ^c^	0.026	<0.001
Upper Ileum ^3^							
TFA	0.74 ^a^	0.68 ^a^	0.53 ^b^	0.35 ^c^	0.51 ^b^	0.027	<0.001
SFA	0.65 ^a^	0.49 ^b^	0.32 ^c^	0.12 ^d^	0.36 ^bc^	0.033	<0.001
MUFA	0.73 ^a^	0.69 ^a^	0.61 ^ab^	0.52 ^b^	0.62 ^ab^	0.031	<0.001
PUFA	0.78 ^a^	0.78 ^a^	0.69 ^ab^	0.59 ^b^	0.63 ^b^	0.028	<0.001
Lower Ileum ^3^							
TFA	0.79 ^a^	0.73 ^a^	0.65 ^b^	0.41 ^c^	0.62 ^b^	0.020	<0.001
SFA	0.69 ^a^	0.55 ^b^	0.47 ^b^	0.18 ^c^	0.49 ^b^	0.022	<0.001
MUFA	0.76 ^a^	0.74 ^a^	0.71 ^a^	0.56 ^b^	0.74 ^a^	0.025	<0.001
PUFA	0.83 ^a^	0.83 ^a^	0.79 ^a^	0.68 ^b^	0.75 ^ab^	0.028	0.002
Excreta ^3^							
TFA	0.80 ^a^	0.73 ^ab^	0.63 ^bc^	0.47 ^d^	0.60 ^c^	0.023	<0.001
SFA	0.64 ^a^	0.53 ^b^	0.43 ^bc^	0.23 ^d^	0.39 ^c^	0.025	<0.001
MUFA	0.79 ^a^	0.77 ^ab^	0.74 ^ab^	0.68 ^b^	0.73 ^ab^	0.021	0.018
PUFA	0.85 ^a^	0.81 ^a^	0.75 ^ab^	0.67 ^b^	0.80 ^a^	0.031	0.004

Abbreviations: AME, apparent metabolizable energy; TFA, total fatty acids; SFA, saturated fatty acids; MUFA, monounsaturated fatty acids; PUFA, polyunsaturated fatty acids. ^1^ Values are pooled means of 6 replicates from chickens fed diets supplemented with 6% of soybean oil (S6), palm fatty acid distillate (PA6), palm oil (P6), or oil blends with 4% soybean oil and 2% palm fatty acid distillate (S4-PA2) or 2% soybean oil and 4% palm fatty acid distillate (S2-PA4). ^2^ Values are pooled means of 6 replicates with 16 chickens/replicate. ^3^ Values are pooled means of 6 replicates with 12 chickens/replicate. ^4^ SEM = standard error of the mean. a–d: means in a row not sharing a common letter are significantly different (*p* < 0.05).

**Table 8:** Feed apparent metabolizable energy values and apparent fatty-acid digestibility coefficients along the intestinal tract and excreta according to different fat sources in the diet in 35-d-old broiler chickens.

Item	Dietary Treatments ^1^					SEM ^4^	*p*-Value
	S6	S4-PA2	S2-PA4	PA6	P6		
AME, kcal/kg ^2^	3364 ^a^	3379 ^a^	3212 ^bc^	3121 ^c^	3279 ^ab^	32.48	<0.001
Upper Jejunum ^3^							
TFA	0.48 ^ab^	0.53 ^a^	0.32 ^ab^	0.29 ^b^	0.51 ^a^	0.052	0.005
SFA	0.21 ^b^	0.46 ^ab^	0.26 ^ab^	0.25 ^ab^	0.48 ^a^	0.062	0.009
MUFA	0.60 ^ab^	0.66 ^ab^	0.52 ^ab^	0.50 ^b^	0.67 ^a^	0.042	0.015
PUFA	0.51^a^	0.49^a^	0.20^bc^	0.06^c^	0.34^ab^	0.063	<0.001
Lower Jejunum ^3^							
TFA	0.81 ^a^	0.78 ^a^	0.65 ^b^	0.62 ^b^	0.69 ^b^	0.021	<0.001
SFA	0.73 ^ab^	0.76 ^a^	0.59 ^c^	0.55 ^c^	0.64 ^bc^	0.031	<0.001
MUFA	0.85 ^a^	0.84 ^a^	0.76 ^b^	0.77 ^b^	0.83 ^a^	0.016	<0.001
PUFA	0.81 ^a^	0.76 ^a^	0.62 ^b^	0.54 ^b^	0.56 ^b^	0.031	<0.001
Upper Ileum ^3^							
TFA	0.89 ^a^	0.89 ^a^	0.77 ^bc^	0.72 ^c^	0.82 ^b^	0.015	<0.001
SFA	0.85 ^a^	0.86 ^a^	0.67 ^bc^	0.61 ^c^	0.77 ^ab^	0.030	<0.001
MUFA	0.91 ^a^	0.91 ^a^	0.85 ^b^	0.84 ^b^	0.91 ^a^	0.008	<0.001
PUFA	0.90 ^a^	0.90 ^a^	0.82 ^b^	0.77 ^c^	0.78 ^c^	0.008	<0.001
Lower Ileum ^3^							
TFA	0.92 ^a^	0.92 ^a^	0.82 ^b^	0.76 ^c^	0.84 ^b^	0.010	<0.001
SFA	0.90 ^a^	0.90 ^a^	0.71 ^c^	0.64 ^d^	0.78 ^b^	0.017	<0.001
MUFA	0.93 ^a^	0.93 ^a^	0.88 ^b^	0.88 ^b^	0.93 ^a^	0.006	<0.001
PUFA	0.93 ^a^	0.93 ^a^	0.87 ^b^	0.85 ^b^	0.83 ^b^	0.013	<0.001
Excreta ^3^							
TFA	0.93 ^a^	0.92 ^a^	0.84 ^b^	0.72 ^c^	0.84 ^b^	0.009	<0.001
SFA	0.87 ^a^	0.87 ^a^	0.76 ^b^	0.59 ^c^	0.77 ^b^	0.016	<0.001
MUFA	0.93 ^a^	0.93 ^a^	0.89 ^b^	0.85 ^c^	0.92 ^a^	0.005	<0.001
PUFA	0.94 ^a^	0.94 ^a^	0.90 ^b^	0.82 ^c^	0.84 ^c^	0.009	<0.001

Abbreviations: AME, apparent metabolizable energy; TFA, total fatty acids; SFA, saturated fatty acids; MUFA, monounsaturated fatty acids; PUFA, polyunsaturated fatty acids. ^1^ Values are pooled means of 6 replicates from chickens fed diets supplemented with 6% of soybean oil (S6), palm fatty acid distillate (PA6), palm oil (P6), or oil blends with 4% soybean oil and 2% palm fatty acid distillate (S4-PA2) or 2% soybean oil and 4% palm fatty acid distillate (S2-PA4). ^2^ Values are pooled means of 6 replicates with 4 chickens/replicate. ^3^ Values are pooled means of 6 replicates with 2 chickens/replicate. ^4^ SEM = standard error of the mean. a–d: means in a row not sharing a common letter are significantly different (*p* < 0.05).

## Supplementary Materials

The following are available online at https://www.mdpi.com/article/10.3390/ani11041035/s1, Table S1: Lipid-class content according to different intestinal segments and excreta in 11- and 35-day-old broiler chickens, Table S2. Lipid-class content in the lower ileum according to different fat sources in the diet in 11- and 35-day-old broiler chickens, Table S3. Feed apparent metabolizable energy value and apparent fatty-acid digestibility coefficients in the lower ileum according to different fat sources in the diet in 11- and 35-day-old broiler chickens, Table S4. Contribution of each intestinal segment to FA absorption according to the age of the chicken.
