# Supplementary material for: Soybean Oil Replacement by Palm Fatty Acid Distillate in Broiler Chicken Diets: Fat Digestibility and Lipid-Class Content along the Intestinal Tract
Source: Animals (Basel). 2021 Apr 6;11(4):1035. doi: 10.3390/ani11041035 (PMC8067497; doi:10.3390/ani11041035)
Supplement: Supplementary file 1 [file animals-11-01035-s001.pdf]

animals

MDPI

Article

Soybean Oil Replacement by Palm Fatty Acid Distillate in Broiler Chicken Diets: Fat Digestibility and Lipid-Class Content along the Intestinal Tract

Beatriz Jimenez-Moya [1], Ana C. Barroeta [1], Alba Tres [2,3], María Dolores Soler [4] and Roser Sala [1,*]

[1] Animal Nutrition and Welfare Service (SNiBA), Animal and Food Science Department, Faculty of Veterinary, Universitat Autònoma de Barcelona, Edifici V, Travessera dels Turons, 08193 Bellaterra, Spain; Beatriz.Jimenez@uab.cat (B.J.-M.); Ana.Barroeta@uab.cat (A.C.B.)
[2] Departament de Nutrició, Ciències de l'Alimentació i Gastronomia, Campus de l'Alimentació Torribera, Facultat de Farmàcia i Ciències de l'Alimentació, Universitat de Barcelona, Av Prat de la Riba, 171, 08921 Santa Coloma de Gramenet, Spain; atres@ub.edu
[3] Institut de Recerca en Nutrició i Seguretat Alimentària (INSA-UB), Universitat de Barcelona, Av Prat de la Riba, 171, 08921 Santa Coloma de Gramenet, Spain
[4] AviFeed Science, Department of Animal Production and Health Public Veterinary Health and Food Science and Technology, Facultad de Veterinaria, Universidad Cardenal Herrera-CEU, CEU Universities, Calle Tirant lo Blanch, 7, 46115 Alfara del Patriarca, Valencia, Spain; mariola@uchceu.es
* Correspondence: Roser.Sala@uab.cat

Simple Summary: Palm fatty acid distillate is a by-product of palm oil refining. It is of both environmental and economic interest to include it in the diets of broiler chickens. However, its high saturation degree and acidity level limit its use. This study aimed to assess the effect of replacing soybean oil with increasing levels of palm fatty acid distillate on the utilization of fat by broilers. Dietary fat hydrolysis was mostly affected by the age of the bird and including palm fatty acid distillate mainly affected the absorption process. The replacement of soybean oil by palm fatty acid distillate reduced the total fat utilization, and in starter chicks delayed the site of fatty acid absorption. As the age increased, the digestibility of saturated fatty acids improved, and, above all, it improved the free fatty acid utilization. Therefore, the potential inclusion of palm fatty acid distillate for broiler feeds depends on the age of the bird. It would not be recommended to include this by-product in starter feeds. However, for the grower-finisher phase, blending palm fatty acid distillate with soybean oil (1:3, w/w) could be a suitable alternative, that does not have negative repercussions for either fatty acid absorption or growth performance.

Citation: Jimenez-Moya, B.; Barroeta, A.C.; Tres, A.; Soler, M.D.; Sala, R. Soybean Oil Replacement by Palm Fatty Acid Distillate in Broiler Chicken Diets: Fat Digestibility and Lipid-Class Content Along the Intestinal Tract. *Animals* **2021**, *11*, 1035. https://doi.org/10.3390/ani11041035

Academic Editor: Beob Gyun Kim

Received: 2 March 2021
Accepted: 1 April 2021
Published: 6 April 2021

Publisher's Note: MDPI stays neutral with regard to jurisdictional claims in published maps and institutional affiliations.

Copyright: © 2021 by the authors. Licensee MDPI, Basel, Switzerland. This article is an open access article distributed under the terms and conditions of the Creative Commons Attribution (CC BY) license (http://creativecommons.org/licenses/by/4.0/).

Table S1. Lipid-class content according to different intestinal segments and excreta in 11- and 35-day-old broiler chickens.

	Intestinal Segment and Excreta [1]					*p*-Values	
**Item**	**Upper Jejunum**	**Lower Jejunum**	**Upper Ileum**	**Lower Ileum**	**Excreta**	**SEM**	**P**
**11-day-old broiler chickens**							
TAG	0.44 [a]	0.35 [ab]	0.26 [b]	0.28 [b]	0.32 [b]	0.022	<0.001
DAG	1.83 [a]	0.88 [bc]	0.68 [bc]	0.63 [c]	1.05 [b]	0.097	<0.001
MAG	0.18 [ab]	0.18 [ab]	0.15 [b]	0.22 [a]	0.16 [b]	0.014	0.004
FFA	15.22 [a]	8.70 [b]	7.67 [b]	7.64 [b]	8.93 [b]	0.913	<0.001
**35-day-old broiler chickens**							
TAG	0.21 [a]	0.22 [a]	0.13 [bc]	0.09 [c]	0.16 [ab]	0.015	<0.001
DAG	1.58 [a]	0.60 [b]	0.18 [c]	0.15 [c]	0.17 [c]	0.062	<0.001
MAG	0.25 [a]	0.20 [ab]	0.13 [c]	0.17 [bc]	0.12 [c]	0.015	<0.001
FFA	10.06 [a]	5.29 [b]	2.60 [c]	2.54 [c]	2.78 [c]	0.417	<0.001

[1] Values are pooled means of 30 replicates from chickens fed diets at 11-day-old (n = 150) or 35-day-old (n = 150). TAG = triacylglycerols; DAG = diacylglycerols; MAG = monoacylglycerols; FFA = free fatty acids. SEM = standard error of the mean. *p*-Values were obtained from univariate ANOVA conducted for each age to study whether the intestinal segment (or excreta) affected the lipid-class content. a–c: means in a row not sharing a common letter are significantly different (p < 0.05).

Table S2. Lipid-class content in the lower ileum according to different fat sources in the diet in 11- and 35-day-old broiler chickens.

	Dietary Treatments [1]										*p*-Values									
	11-day-old broiler chickens					35-day-old broiler chickens					S6		S4-PA2		S2-PA4		PA6		P6	
**Item**	**S6**	**S4-PA2**	**S2-PA4**	**PA6**	**P6**	**S6**	**S4-PA2**	**S2-PA4**	**PA6**	**P6**	**SEM**	**P**	**SEM**	**P**	**SEM**	**P**	**SEM**	**P**	**SEM**	**P**
TAG	0.32	0.29	0.27	0.33	0.19	0.09	0.11	0.09	0.07	0.07	0.020	<0.001	0.044	0.015	0.029	0.001	0.051	0.006	0.021	0.002
DAG	0.64	0.60	0.76	0.71	0.45	0.14	0.17	0.11	0.18	0.17	0.069	<0.001	0.052	<0.001	0.072	<0.001	0.032	<0.001	0.071	0.021
MAG	0.23	0.25	0.25	0.23	0.15	0.13	0.14	0.21	0.21	0.15	0.014	<0.001	0.036	0.051	0.027	0.384	0.027	0.483	0.019	1.000
FFA	3.02	5.23	8.78	12.70	8.47	0.87	1.32	2.77	4.82	2.92	0.252	<0.001	0.290	<0.001	0.443	<0.001	0.212	<0.001	0.574	<0.001

[1]Values are pooled means of 6 replicates from chickens fed diets supplemented with 6% of soybean oil (S6), palm fatty acid distillate (PA6), palm oil (P6), or oil blends with 4% soybean oil and 2% palm fatty acid distillate (S4-PA2) or 2% soybean oil and 4% palm fatty acid distillate (S2-PA4). TAG = triacylglycerols; DAG = diacylglycerols; MAG = monoacylglycerols; FFA = free fatty acids. SEM = standard error of the mean. *p*-Values were obtained from univariate ANOVA conducted for each dietary treatment to study whether the age affected the lipid-class content ($n = 12$). $p < 0.05$ was considered significant.

Table S3. Feed apparent metabolizable energy value and apparent fatty-acid digestibility coefficients in the lower ileum according to different fat sources in the diet in 11- and 35-day-old broiler chickens.

	Dietary Treatments [1]										*p*-Values									
	11-day-old broiler chickens					35-day-old broiler chickens														
											S6		S4-PA2		S2-PA4		PA6		P6	
**Item**	**S6**	**S4-PA2**	**S2-PA4**	**PA6**	**P6**	**S6**	**S4-PA2**	**S2-PA4**	**PA6**	**P6**	**SEM**	**P**	**SEM**	**P**	**SEM**	**P**	**SEM**	**P**	**SEM**	**P**
AME, kcal/kg [2]	3348	3340	3074	2760	3014	3364	3379	3212	3121	3279	24.64	0.656	30.21	0.381	23.86	0.002	35.64	<0.001	31.27	<0.001
FA digestibility																				
TFA	0.79	0.73	0.65	0.41	0.62	0.92	0.92	0.82	0.76	0.84	0.011	<0.001	0.011	<0.001	0.019	<0.001	0.012	<0.001	0.022	<0.001
SFA	0.69	0.55	0.47	0.18	0.49	0.90	0.90	0.71	0.64	0.78	0.011	<0.001	0.023	<0.001	0.018	<0.001	0.016	<0.001	0.026	<0.001
MUFA	0.76	0.74	0.71	0.56	0.74	0.93	0.93	0.88	0.88	0.93	0.010	<0.001	0.011	<0.001	0.022	<0.001	0.012	<0.001	0.026	<0.001
PUFA	0.83	0.83	0.79	0.68	0.75	0.93	0.93	0.87	0.85	0.83	0.011	<0.001	0.011	<0.001	0.026	**0.050**	0.021	<0.001	0.028	**0.056**

[1]Values are pooled means of 6 replicates from chickens fed diets supplemented with 6% of soybean oil (S6), palm fatty acid distillate (PA6), palm oil (P6), or oil blends with 4% soybean oil and 2% palm fatty acid distillate (S4-PA2) or 2% soybean oil and 4% palm fatty acid distillate (S2-PA4). AME = apparent metabolizable energy, TFA = total fatty acids, SFA = saturated fatty acids, MUFA = monounsaturated fatty acids, PUFA = polyunsaturated fatty acids, SEM = standard error of the mean. *p*-Values were obtained from univariate ANOVA conducted for each dietary treatment to study whether the age affected the feed AME values, and the FA digestibility results ($n = 12$). $p < 0.05$ was considered significant.

Table S4. Contribution of each intestinal segment to FA absorption according to the age of the chicken.

	Age (days) 1		p–Value	
**Item**	**11**	**35**	**SEM**	**Age**
**Upper jejunum**				
TFA	48.25	45.93	5.542	0.577
Palmitic	41.33	49.70	4.742	0.234
Stearic	-	-	-	-
Oleic	51.71	60.56	4.272	0.168
Linoleic	47.50	35.14	6.472	0.138
**Lower jejunum**				
TFA	36.24	37.85	5.09	0.735
Palmitic	39.38	36.87	4.753	0.521
Stearic	52.89	65.55	4.783	0.069
Oleic	33.00	29.31	3.957	0.342
Linoleic	37.68	40.06	5.283	0.872
**Upper ileum**				
TFA	5.52	12.36	1.299	<0.001
Palmitic	6.40	9.55	1.755	0.293
Stearic	18.80	24.73	3.863	0.380
Oleic	7.27	7.50	1.076	0.999
Linoleic	6.92	19.44	1.683	<0.001
**Lower ileum**				
TFA	9.99	3.86	1.206	<0.001
Palmitic	12.89	3.88	1.882	<0.001
Stearic	28.32	9.72	3.590	<0.001
Oleic	8.02	2.62	1.040	<0.001
Linoleic	7.90	5.36	0.984	0.089

[1]Values are means of 30 replicates from chickens fed diets at 11-day-old or 35-day-old. TFA = total fatty acids. SEM = standard error of the mean. *p*-Values were obtained from univariate ANOVA conducted for each intestinal segment to study whether the age affected the FA absorption ($n = 60$). $p < 0.05$ was considered significant.
